# Effect of anthelmintic treatment on serum free IGF-1 and IGFBP-3: a cluster-randomized-controlled trial in Indonesia

**DOI:** 10.1038/s41598-020-75781-4

**Published:** 2020-11-04

**Authors:** Farid Kurniawan, Dicky L. Tahapary, Karin de Ruiter, Em Yunir, Nienke R. Biermasz, Johannes W. A. Smit, Taniawati Supali, Erliyani Sartono, Maria Yazdanbakhsh, Pradana Soewondo

**Affiliations:** 1 Division of Endocrinology and Metabolism, Department of Internal Medicine, Cipto Mangunkusumo National General Hospital/Faculty of Medicine Universitas Indonesia, Jl. Diponegoro No. 71, Jakarta Pusat, DKI Jakarta 10430 Indonesia; 2 Metabolic, Cardiovascular, and Aging Research Cluster, The Indonesian Medical Education and Research Institute, Faculty of Medicine Universitas Indonesia, Jakarta, Indonesia; 3 Department of Parasitology, Leiden University Medical Center, Leiden, The Netherlands; 4 Department of Endocrinology and Metabolic Disease, Leiden University Medical Center, Leiden, The Netherlands; 5 Department of Internal Medicine, Radboud University Medical Center, Nijmegen, The Netherlands; 6 Department of Internal Medicine, Leiden University Medical Center, Leiden, The Netherlands; 7 Department of Parasitology, Faculty of Medicine Universitas Indonesia, Jakarta, Indonesia

**Keywords:** Endocrinology, Endocrine system and metabolic diseases, Pathogenesis, Infection

## Abstract

In children, soil-transmitted helminth infections have been linked to poor nutritional status and growth retardation in association with lower levels of IGF-1. In adults, IGF-1 has an anabolic and metabolic function and is related to nutritional status. Here, we assessed the impact of helminth infection on free IGF-1 and its major binding protein, IGFBP-3, in adults. The levels of IGF-1 and IGFBP3 were measured in 1669 subjects aged ≥ 16 years, before and after receiving four rounds of albendazole 400 mg/day or matching placebo for three consecutive days. Helminth infection status was assessed by microscopy (Kato-Katz) and PCR. Serum free IGF-1 level was significantly lower in helminth-infected subjects [mean difference and 95% CI − 0.068 (− 0.103; − 0.033), P < 0.001 after adjustment for age, sex, body mass index, and fasting insulin level]. There was no difference in IGFBP-3 level between helminth infected versus non-infected subjects. In the whole study population, albendazole treatment significantly increased serum free IGF-1 level [estimate and 95% CI 0.031 (0.004; − 0.057), P = 0.024] whereas no effect was found on the IGFBP-3 level. Our study showed that helminth infection in adults is associated with lower free IGF-1 levels but not with IGFBP-3 and albendazole treatment significantly increases free IGF-1 levels in the study population.

**Clinical Trial Registration**: https://www.isrctn.com/ISRCTN75636394.

## Introduction

Soil-transmitted helminth (STH) infections are still highly prevalent, especially in low- to middle-income countries^1^. In 2010, more than 1.6 billion people were estimated to be infected with at least one of the three main STH species [*Ascaris lumbricoides*, hookworms (*Ancylostoma duodenale* and *Necator americanus*), and *Trichuris trichiura*]^2^. In Indonesia, overall, around 22% of the population were infected with *Ascaris*, 20% with hookworm, and 12% with *Trichuris*. The prevalence could be higher in certain region of Indonesia, especially in rural areas^3^. STH infections have been associated with adverse effects on health in children, such as malnutrition and growth disorders^4^. Conversely, in adults, STH infections have been associated with a lower insulin resistance (IR)^5^, thus indicating a possible beneficial effect by counterbalancing modern health threats such as metabolic syndrome^6^.

Insulin-like growth factor-1 (IGF-1) is a hormone mainly produced by the liver under the influence of growth hormone (GH) from the pituitary gland. Most of circulating IGF-1 are bound to its six binding proteins with IGF-binding protein-3 (IGFBP-3) being the major one (> 75%). Only IGF-1 in the free-form is considered biologically active^7^. IGF-I has important role on musculoskeletal growth and development^8,9^, cell proliferation^10^ and tissue repair^11^, through its binding to IGF-I receptors and activation of the Akt/protein kinase-B pathway^12^. Because of its structural homology with proinsulin^13^, IGF-1 also binds to the insulin receptor although with much lower affinity than insulin and exerts its metabolic properties^14^. These actions include promotes glucose uptake and transport in certain peripheral tissues^15^, modulates glycogen synthesis^16^, promotes fatty acid transport^17^, and regulates amino acid and glucose intestinal absorption^18,19^. Additionally, IGF-1 concentrations are dependent on nutritional state and decrease during calorie and protein restriction^20^. Inflammation could also significantly affect IGF-1 levels as IGF-1 values have been found to be reduced during systemic inflammation^21^.

As helminth infections affect both nutritional and inflammatory status^22^, they could also affect IGF-1 concentration. A study in children has indeed shown that *Trichuris*-mediated dysentery syndrome was associated with lower IGF-1 levels and was related to the observed growth disorder^23^. It is possible that STH infections also affect IGF-1 levels in adults. To address this, we conducted a study in the adult population living in an area endemic for helminth infection to evaluate the association between STH infections and free IGF-1 concentration as well as its main binding protein, IGFBP-3. Subsequently, to assess causality, we investigated the effect of anthelmintic treatment on serum free IGF-1 and IGFBP-3 levels to evaluate the effect of helminth infections on IGF system.

## Results

### Study population

At baseline, there were 1669 subjects recruited in the SUGARSPIN trial. After exclusion of 65 subjects (36 and 29 subjects from placebo and albendazole arms, respectively) with insufficient serum samples, a total of 1604 subjects were included in the analysis. At the follow-up time point, there were 1295 serum samples available for analysis because of incomplete follow up data and loss to follow-up due to death, moving away from the study area, and refusal to continue participation in the study (see Consort Diagram in Supplementary Fig. 1).

Baseline characteristics for both treatment arms were similar. Majority of the study participants were female (61.8% vs 59.1%, for placebo and albendazole groups, respectively). Mean BMI in both genders were also similar between the two treatment arms (male: 21.8 vs. 21.9 kg/m^2^; female: 22.9 vs. 22.7 kg/m^2^, for placebo and albendazole groups, respectively). In addition, the levels of serum fasting insulin, free IGF-1, IGFBP-3, and hsCRP were also comparable between two groups. Based on microscopy results, at baseline 44.3% of the subjects in the placebo and 42.0% in the albendazole arms were infected with STH and when PCR was used for detection of parasites, again helminth infection prevalence was similar in the two arms (54.6% vs. 54.7% for placebo and albendazole arms, respectively). Proportion of subjects with single or multiple infections were also similar in both placebo and albendazole arms (Table 1).

**Table 1:** Baseline characteristics.

	Placebo N = 836	Albendazole N = 768
Sex (n male, %)	319 (38.2)	308 (40.1)
Age (mean in years, SD)		
Male	44.45 (15.87)	42.91 (15.73)
Female	40.90 (15.21)	42.29 (15.63)
BMI (kg/m^2^) (mean, SD)		
Male	21.81 (3.81)	21.91 (3.58)
Female	22.90 (4.33)	22.73 (4.21)
Fasting insulin^a^ (mU/L)	3.45 (3.22–3.73)	3.52 (3.27–3.79)
Free IGF-1^a^ (ng/mL)	0.359 (0.337–0.382)	0.369 (0.345–0.395)
Proportion of free IGF-1 below detection limit (%, n/N)	35.9 (300/836)	35.8 (275/768)
IGFBP-3^a^ (ng/mL)	424.75 (415.94–433.74)	423.73 (414.17–433.51)
hsCRP^a^ (mg/L)	0.96 (0.89–1.04)	0.97 (0.90–1.06)
Helminth infection by microscopy (%infected, n/N)	44.2 (347/785)	42.0 (300/714)
Number of helminth infection by microscopy (%infected, n/N)		
1 species	27.8 (218/785)	26.8 (191/714)
2 species’	11.3 (89/785)	11.6 (83/714)
3 species’	5.1 (40/785)	3.6 (26/714)
Helminth infection by PCR (%infected, n/N)	54.6 (412/754)	54.7 (375/686)
Number of helminth infection by PCR (%infected, n/N)		
1 species	32.9 (248/754)	35.4 (243/686)
2 species’	16.7 (126/754)	14.7 (101/686)
3 species’	5.0 (38/754)	4.5 (31/686)

*BMI* body mass index, *IGF-1* insulin-like growth factor-1, *IGFBP-3* insulin-like growth factor binding protein-3.

^a^Non-normally distributed continuous variables, presented as geomean (95% CI).

### Baseline free IGF-1 and IGFBP-3 levels as a function of helminth infection

**Figure 1:**
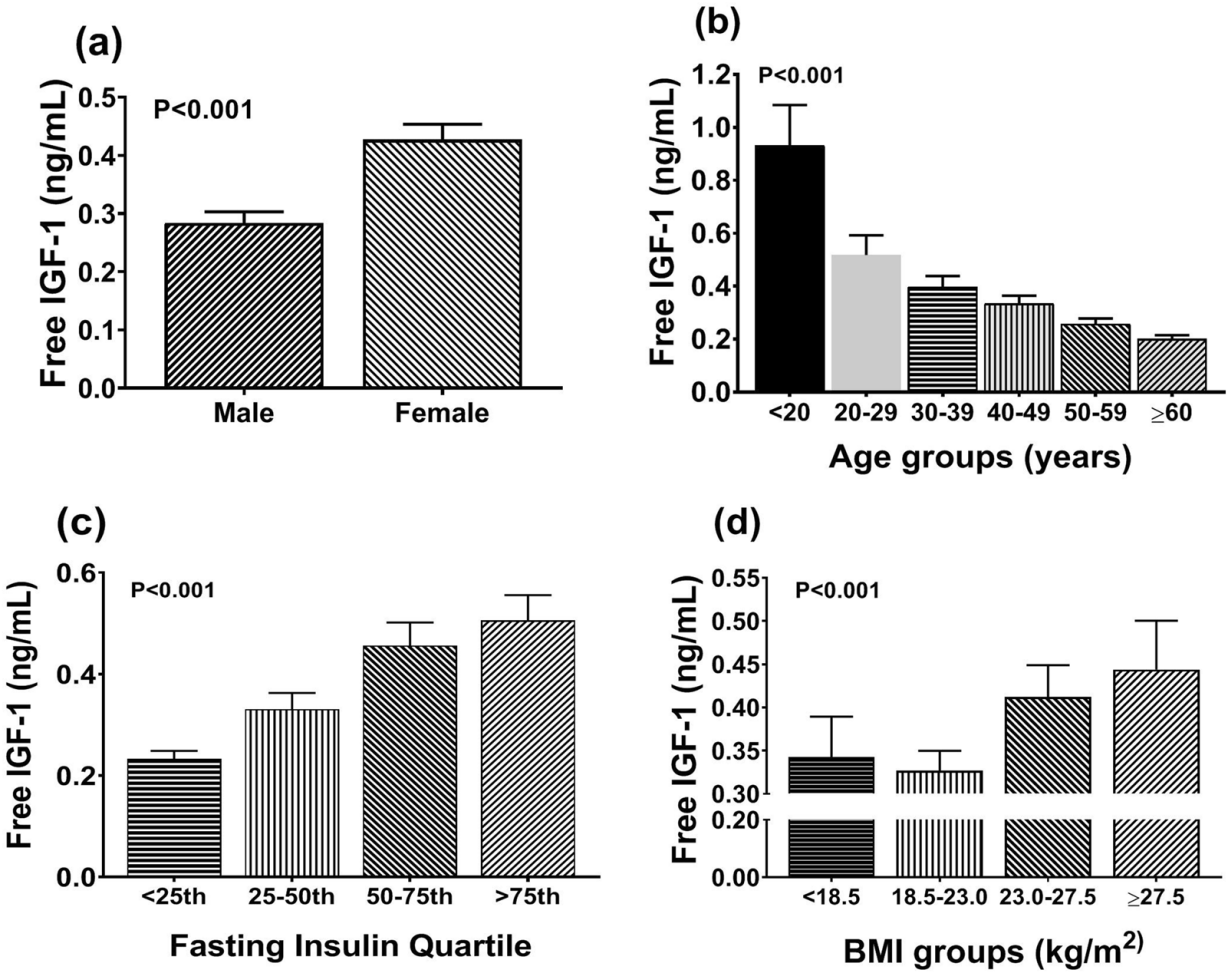
Serum free IGF-1 in males and females (**a**) and the association between IGF-1 and -age groups (**b**), -fasting insulin quartiles (**c**), and -BMI groups (**d**). Serum free IGF-1 level was presented as geometric mean and its 95% confidence interval. Serum free IGF-1 and fasting insulin levels are log transformed for analysis. BMI grouping was based on WHO classification for Asian population. (N for sex, age, fasting insulin quartiles grouping = 1604 subjects; N for BMI grouping = 1597 subjects).

Male subjects had lower level of serum free IGF-1 compared with female subjects [mean difference (95% CI) − 0.178 (− 0.216; − 0.138), P < 0.001, Fig. 1a]. As expected, serum free IGF-1 levels decline with increasing age [− 0.121 (− 0.132; − 0.109), P < 0.001, Fig. 1b], but increased with increasing fasting insulin levels [0.087 (0.071; 0.104), P < 0.001 in age, sex and BMI adjusted model, Fig. 1c]. In age and sex adjusted models, the levels of IGF-1 were also increased with increasing BMI [0.055 (0.038; 0.071), P < 0.001, Fig. 1d].

When using PCR to detect helminth infection, we found a significantly lower serum free IGF-1 among STH-infected compared to uninfected subjects [− 0.090 (− 0.132; − 0.048), P < 0.001] and this did not change following adjustment with age and sex [− 0.89 (− 0.125; − 0.053), P < 0.001]. The mean differences, following adjustment with BMI [− 0.078 (− 0.115; − 0.042), P < 0.001] as well as a further adjustment with fasting insulin [− 0.068 (− 0.103; − 0.033), P < 0.001], were slightly attenuated (Table 2a). As inflammation is considered to be an important factor in assessing IGF-1 levels, we used hsCRP as a proxy for inflammation. Adjustment for hsCRP did not change the results, which still showed lower free IGF-1 levels in STH infected compared to uninfected groups [− 0.071 (− 0.107; − 0.036), P < 0.001)]. Regarding IGFBP-3, no differences were found between STH-infected and uninfected subjects [0.001 (− 0.014; 0.015), P = 0.937].

**Table 2:** Association between free IGF-1 or IGFBP-3 with STH infection status based on PCR (a) and microscopy (b).

		STH-infected	STH non-infected	Un-adjusted	Adjusted for age and sex	Adjusted for age, sex, and BMI	Adjusted for age, sex, BMI, and fasting insulin
(a)	Free IGF-1 (ng/mL)	0.33 (0.31–0.35)	0.41 (0.38–0.44)	− 0.090 (− 0.132; − 0.048), P < 0.001	− 0.089 (− 0.125; − 0.053), P < 0.001	− 0.078 (− 0.115; − 0.042), P < 0.001	− 0.068 (− 0.103; − 0.033), P < 0.001
	IGFBP-3 (ng/mL)	424.50 (415.38–433.81)	423.94 (413.59–434.54)	0.001 (− 0.014; 0.015), P = 0.937			
(b)	Free IGF-1 (ng/mL)	0.35 (0.33–0.38)	0.37 (0.35–0.400)	− 0.027 (− 0.069; − 0.015), P = 0.202	− 0.042 (− 0.078; − 0.006), P = 0.022	− 0.033 (− 0.068; − 0.003), P = 0.074	− 0.031 (− 0.066; − 0.003), P = 0.075
	IGFBP-3 (ng/mL)	422.67 (412.79–432.79)	424.66 (415.51–434.01)	− 0.002 (− 0.016; 0.012), P = 0.777			

All variables are presented as geometric mean and its 95% confidence interval and were log transformed for analysis. Analysis for the difference between STH-infected and non-infected subjects was performed using linear regression and presented as estimated mean difference and its 95% confidence interval.

*BMI* body mass index, *IGF-1* insulin-like growth factor-1, *IGFBP-3* insulin-like growth factor binding protein-3, *STH* soil-transmitted helminth.

When helminth infection was detected based on microscopy, a less sensitive method that misses infection in 10.3% of the subjects, the difference in the level of free IGF-1 and IGFBP-3 between STH-infected and non-infected subjects fell short of statistical significance (Table 2b).

**Figure 2:**
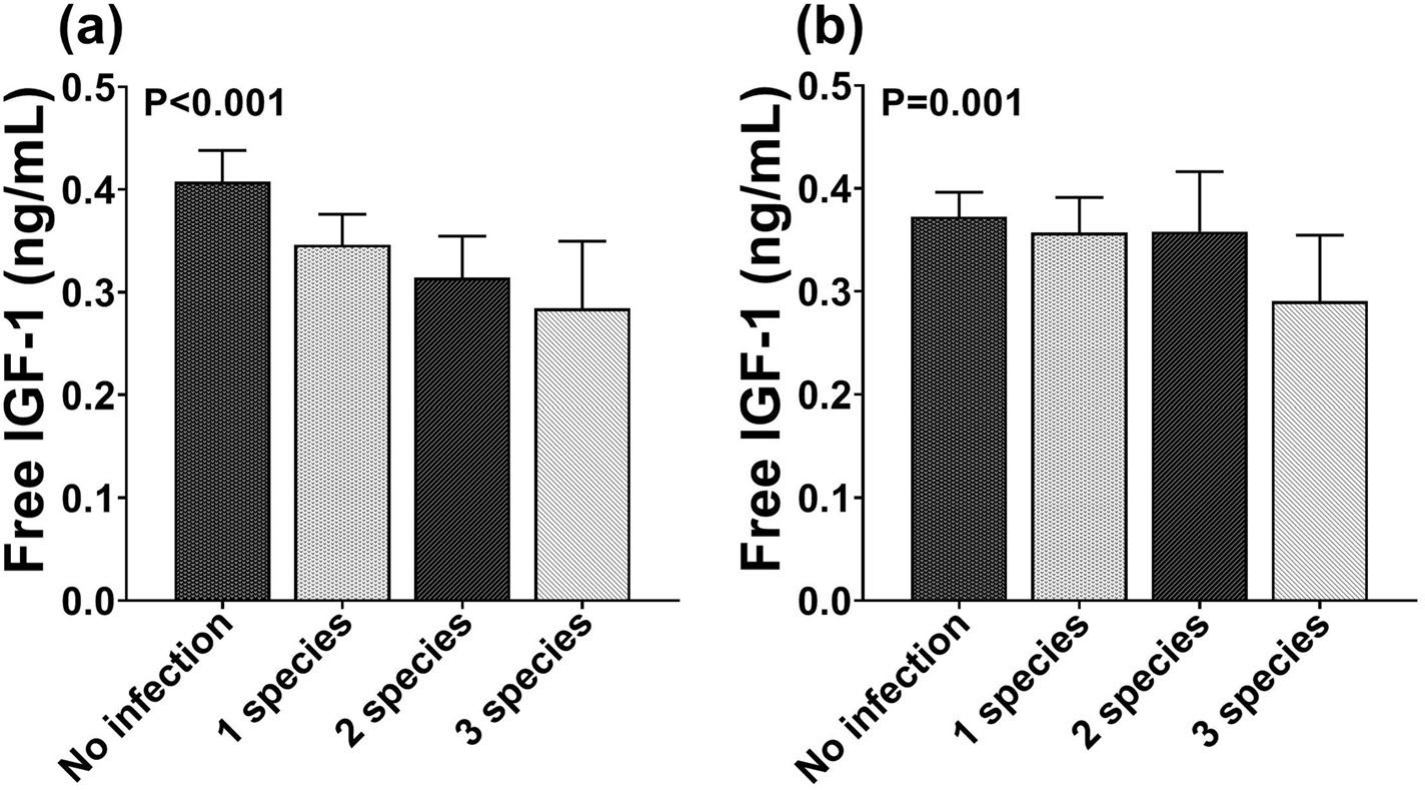
Serum free IGF-1 level based on the number of helminth species detected by PCR (**a**) and microscopy (**b**). Serum free IGF-1 level was presented as geometric mean and its 95% confidence interval and were log-transformed for analysis. (N for PCR = 1440 subjects; N for microscopy = 1499 subjects).

Interestingly, we also observed a decline in the levels of IGF-1 with increasing number of helminth infections, which was more pronounced when infection was detected by PCR [mean difference (95% CI) − 0.044 (− 0.064; − 0.024), P < 0.001 after adjustment for age, sex, BMI, and fasting insulin level) compared to by microscopy [− 0.033 (− 0.053; − 0.013, P = 0.001] (see also Fig. 2a,b).

### Effect of anthelmintic treatment on serum free IGF-1 and IGFBP-3 levels

As reported in the main study^24^, 1 year of albendazole treatment significantly reduced the prevalence of helminth infections, either assessed by PCR (from 54.7 to 28.9%) or microscopy (from 43.2 to 20.2%) (Supplementary Fig. 2).

**Figure 3:**
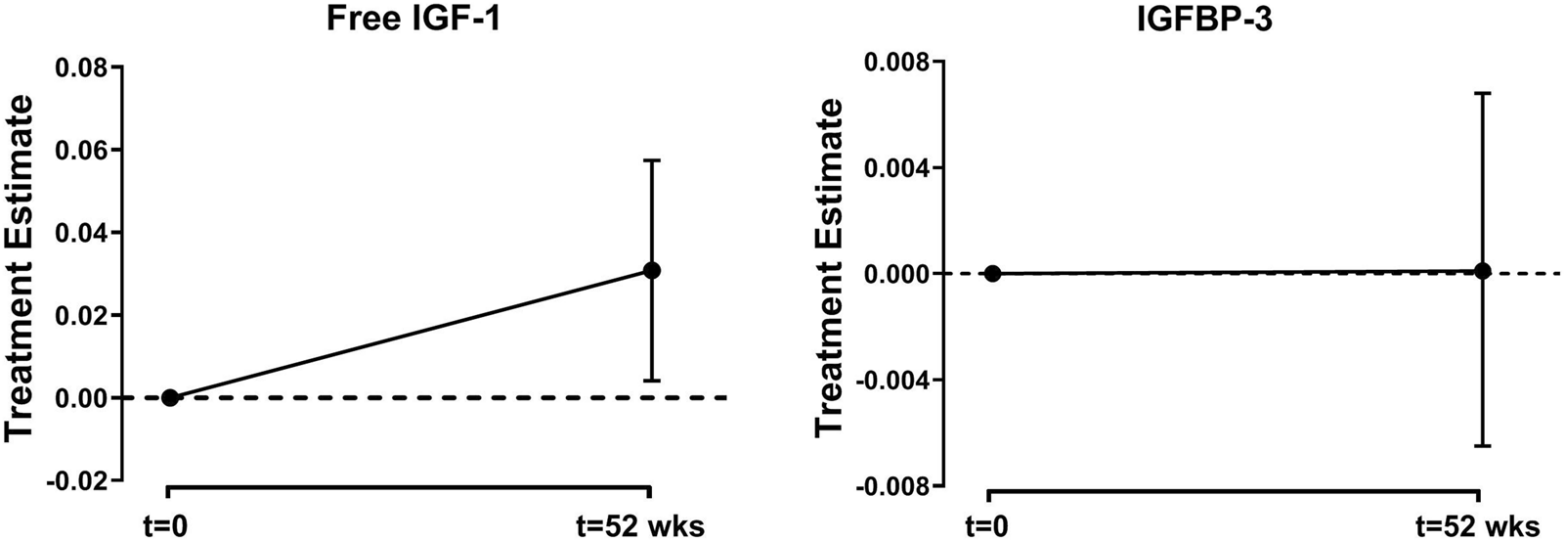
Effect of albendazole treatment on serum free IGF-1 and IGFBP-3 in the whole study population. The estimated treatment effect was obtained using linear mixed model and presented with its corresponding 95% confidence interval.

In the whole study population, albendazole treatment resulted in an increase of serum free IGF-1 [estimate (95% CI) 0.031 (0.004; 0.057), P = 0.024] but not IGFBP-3 levels [0.0001 (− 0.0065; 0.0068), P = 0.968] (Fig. 3). The treatment effect on serum free IGF-1 remained intact following adjustment with BMI [0.030 (0.003; 0.057), P = 0.028] or additional adjustment with fasting insulin [0.029 (0.002; 0.056), P = 0.033].

When the population was stratified into helminth infected and uninfected at baseline to assess the effect of treatment in the two groups, no statistically significant treatment effect on free IGF-1 level was observed, either in the infected, [0.032 (− 0.006; 0.070), P = 0.084 when categorized by PCR, and 0.027 (− 0.015; 0.069), P = 0.07, when categorized by microscopy] or the uninfected groups [0.031 (− 0.092; 0.071), P = 0.130 and 0.035 (− 0.0004; 0.070), P = 0.053; for PCR and microscopy, respectively].

## Discussion

In this study, we observed a significantly lower serum free IGF-1, but not IGFBP-3 level, among STH-infected subjects. Intensive anthelmintic treatment significantly increased serum free IGF-1 level in the adult population studied.

The lower level of free IGF-1 in STH-infected compared to non-infected subjects observed in our study confirmed previous reports in children^23,25^, that showed a lower total IGF-1 level among helminth-infected subjects.

There are several possibilities as to how helminth infection could decrease serum free IGF-1 level. Firstly, calories and/or protein intake could be significantly affected by helminth infection^26^. IGF-1, as well as insulin, are anabolic hormones that promote energy storage and protein synthesis, especially in nutrient sufficient conditions^27^. Under a calorie or protein-restricted condition, a switch to catabolic state takes place by decreasing IGF-1 and insulin secretion with compensatory increased secretion of GH and cortisol, to ensure sufficient substrates like glucose or fatty acids are available for basal metabolism^28^. It is known that under calorie or protein restricted condition, there is increasing GH but the IGF-1 levels remain low due to hepatic resistance against GH^29^. Thus, helminth infection could be seen as a model of long term calories and/or protein restriction in humans. Successful anthelmintic treatment might be considered as a way of nutrient uptake restitution.

The effect of helminth infection on lower free IGF-1 level seemed to be partly mediated through BMI, as adjustment for BMI, attenuated the differences between two groups although remaining significant. In line with previous studies^5,26^, our study also shows that helminth infection is associated with lower BMI and fat mass (data not shown), which in turn can be associated with lower adipose tissue mass. As human adipose tissue can secrete considerable amounts of IGF-1^30,31^, in helminth infected subjects, the lower adipose tissue mass can result in lower IGF-1 levels.

Another mechanism by which helminth infection could affect serum free IGF-1 levels might be mediated by insulin. A lower level of insulin in helminth infection could lead to an increase in IGFBP-1 and -2 levels which eventually will result in a lower level of free IGF-1^31,32^. Our results, which show that, although remaining significant, there is attenuation of the mean differences on free IGF-1 level between STH-infected and non-infected groups after adjustment with fasting insulin level, support this hypothesis.

Helminth infections have been reported to affect the immune system by shifting the phenotype of T cells towards the CD4 + T helper type 2 (TH2) cells and T regulatory (Treg) and reduction in TH1 phenotypes thereby, reduction in its associated pro-inflammatory responses^33^. As previous studies have shown that inflammation could significantly influence the IGF-1 level^21^, adjustment for hsCRP as an inflammatory marker was also performed in this study albeit no significant changes was observed in the differences between helminth infected and uninfected subjects. This is in line with the general concept that chronic helminth infections have no inflammatory but rather an anti-inflammatory impact^34^ and shows that the effect of helminth infection on IGF-1 is not mediated by inflammation.

Previous animal and in-vitro studies have shown that IGF-1 could induce the secretion of interleukin (IL)-10 from immune cells^35,36^ However, the observed lower free IGF-1 level in our current study was not in line with the increase of IL-10 level usually observed during helminth infections due to activation of TH2 and Treg cells. Thus, it will be interesting to study the interaction between IGF-1 and IL-10 in helminth infected subjects.

Finally, several studies have shown that STH infections affect gut microbiota composition^37^. There is recent evidence that the gut microbiota can influence the production of IGF-1^38^. Therefore, it remains possible that STH infections might have their effect on serum free IGF-1 levels via modulation of the gut microbiota.

The observation regarding decreasing levels of IGF-1 with an increasing number of helminth infections in this study might suggest more disruption of nutrient intake, which in turn could directly or indirectly be linked to changes in the BMI and fasting insulin level.

In this study, we found a significant increase of serum free IGF-1 level in the whole population after albendazole treatment, even after adjustment for BMI or fasting insulin levels. However, treatment effect on STH-infected and non-infected subjects seemed largely similar. These might suggest that albendazole has a broader effect than only on STH. It could affect the gut microbiome^39^, thus lead to the changes in IGF-1 levels after treatment. Additionally, intestinal protozoa, which have close interaction with gut microbiome^40^, could be significantly affected by albendazole treatment^41^. This might indirectly affect IGF-1 levels. Another reason for this finding might be because the SUGARSPIN trial was not originally designed for this study. From the original study result, we could observe that albendazole treatment significantly increased insulin resistance in helminth-infected but not in the helminth-uninfected subjects^24^. The complexity of the IGF system and the fact that insulin is also considered as a part of this system^42^, might explain the difference in our study findings compared to the original study.

This is the first human study that evaluated the effect of STH infection and the longitudinal assessment after anthelmintic therapy on serum levels of free IGF-1 and IGFBP-3 in adults. The relatively large number of study subjects and the randomized double-blind study design of anthelmintic treatment can be considered as the strength of this study. However, there are still some limitations. For example, measurement of IGFBP-1, GH, and gut microbiome could have helped to get a clearer picture of the effect of STH infections and anthelmintic treatment on the whole IGF-system.

In conclusion, STH infections were associated with a significantly lower serum free IGF-1 level, which was partly explained by the lower BMI and a lower fasting insulin level but not inflammatory status. At population level, albendazole treatment increases serum free IGF-1 levels but not IGFBP-3. Further studies are needed to disentangle the complex mechanisms underlying the association between helminths and IGF-1 levels in adults.

## Material and methods

### Study design

This present study was part of the SUGARSPIN trial, which has been described previously^43^. Briefly, we conducted a household-based cluster-randomized double blind anthelminthic trial in Flores Island, Indonesia, a region endemic for STH infections. The population was randomized using computer aided block randomization at household level, utilizing Random Allocation Software to assign treatment groups. After randomization, all subjects received three-monthly, of either a single dose of albendazole (400 mg) or matching placebo treatment for three consecutive days. This treatment regimen was given every three months for a total of four rounds^43^. Both study investigators and subjects were blinded to treatment codes. The treatment code was unblinded when all data needed for analysis were cleaned and entered into the database. The study was approved by the ethics committee of Faculty of Medicine, Universitas Indonesia (FKUI) (ref: 549/H2·F1/ETIK/2013), filed by the ethics committee of Leiden University Medical Center (LUMC), and performed in accordance with the principles of the revised Declaration of Helsinki. The trial is registered as a clinical trial (https://www.isrctn.com/ISRCTN75636394). All participants in this study had signed the informed consent.

All measurements and sample collections were performed during the first 8 weeks before the start of the first treatment round (baseline) and 6 weeks after the end of the last treatment round (follow-up). Clinical measurements and blood sample collections were performed after an overnight fast, as described previously^43^. Anthropometric measurements of body weight (SECA Model 876, Seca Gmbh Co, Hamburg, Germany) and height (SECA Model 213, Seca Gmbh Co, Hamburg, Germany,) were performed, of which body mass index (BMI) was then calculated.

### Laboratory measurements

Quantification of serum free IGF-1 and IGFBP-3 was performed by enzyme-linked immunosorbent assays (ELISA) using commercial reagents (DuoSet ELISA R&D System Europe Ltd, Abingdon, UK). The standard range was 31.25–2000 pg/mL for free IGF-1 assay (CVa 5.5%) and 125–8000 pg/mL for IGFBP-3 assay (CVa 10.6%). For IGF-1, the level below detection limit of the assay is assigned a value of 0.15 ng/mL. Fasting serum insulin was measured using a solid phase, enzyme-labeled chemiluminescent immunometric assay (Siemens IMMULITE 2000Xpi systems), with the measuring range of 2–300 mU/L (CVa < 7% at all levels). A latex enhanced immunoturbidimetric method was used to measured high-sensitivity C-reactive protein (hsCRP) on Roche Modular P-instrumentation, the measuring range being 0.1–20.0 mg/L.

Identification of STH infection [hookworm (*Necator americanus, Ancylostoma duodenale*), *Ascaris lumbricoides*, *Trichuris trichiura*, *Strongyloides stercoralis*] was assessed using microscopy (Kato Katz) and PCR on stool samples as described elsewhere^44^.

### Statistical analysis

Continuous variables with normal distribution were presented as mean and its standard deviation [mean (SD)]. Non-normally distributed data were presented as geometric mean and its 95% confidence interval [geomean (95% CI)] and were log-transformed for analysis.

We performed a linear regression on baseline data (IBM SPSS Statistics Version 23) to compare the difference in serum free IGF-1 and IGFBP-3 levels between STH-infected and uninfected subjects or between single vs multiple helminth infections. These differences were presented as mean differences (95% CI) and P-value.

Meanwhile, to evaluate the effect of anthelminthic treatment on serum free IGF-1 and IGFBP-3 level, linear mixed models [lme4 package (R software)] to account for the correlation within households were used, as described previously^43^. Two random effects were used: to model clustering within households, a random household specific intercept was used and to model correlation within subjects, a random subject-specific intercept was used. Treatment effect estimates and 95% confidence interval were reported, while P-value was generated from likelihood ratio test comparing the model with and without the treatment effect.

## Supplementary information

Supplementary Figures.

## Data Availability

The datasets generated and analyzed in the current study are available from the corresponding author on reasonable request.

## References

[1] Bethony J (2006). Soil-transmitted helminth infections: Ascariasis, trichuriasis, and hookworm. Lancet.

[2] Pullan RL, Smith JL, Jasrasaria R, Brooker SJ (2014). Global numbers of infection and disease burden of soil transmitted helminth infections in 2010. Parasit. Vectors..

[3] Silver ZA (2018). Geographical distribution of soil transmitted helminths and the effects of community type in South Asia and South East Asia—A systematic review. PLoS Negl. Trop. Dis..

[4] Partners for Parasite Control. Meeting (3rd, 2004: Geneva, Switzerland) & World Health Organization. Strategy Development and Monitoring for Parasitic Diseases and Vector Control Team. Deworming for health and development: report of the third global meeting of the partners for parasite control. *World Health Organization*. https://apps.who.int/iris/handle/10665/69005 (2005).

[5] Wiria AE (2015). Infection with soil-transmitted helminths is associated with increased insulin sensitivity. PLoS ONE.

[6] Tracey EF, McDermott RA, McDonald MI (2016). Do worms protect against the metabolic syndrome? A systematic review and meta-analysis. Diabetes Res. Clin. Pract..

[7] Rosen CJ (1999). Serum insulin-like growth factors and insulin-like growth factor-binding proteins: Clinical implications. Clin. Chem..

[8] Yakar S (2002). Circulating levels of IGF-1 directly regulate bone growth and density. J. Clin. Investig..

[9] Ascenzi F (2019). Effects of IGF-1 isoforms on muscle growth and sarcopenia. Aging Cell.

[10] Huat TJ, Khan AA, Pati S, Mustafa Z, Abdullah JM, Jaafar H (2014). IGF-1 enhances cell proliferation and survival during early differentiation of mesenchymal stem cells to neural progenitor-like cells. BMC Neurosci..

[11] Provenzano PP (2007). Systemic administration of IGF-I enhances healing in collagenous extracellular matrices: Evaluation of loaded and unloaded ligaments. BMC Physiol..

[12] Hakuno F, Takahashi S (2018). 40 years of IGF1: IGF1 receptor signaling pathways. J. Mol. Endocrinol..

[13] Rinderknecht E, Humbel RE (1978). The amino acid sequence of human insulin-like growth factor I and its structural homology with proinsulin. J. Biol. Chem..

[14] Clemmons DR (2012). Metabolic actions of IGF-1 in normal physiology and diabetes. Endocrinol. Metab. Clin. N. Am..

[15] Russell-Jones DL (1995). A comparison of the effects of IGF-I and insulin on glucose metabolism, fat metabolism and the cardiovascular system in normal human volunteers. Eur. J. Clin. Investig..

[16] Muhic M, Vardjan N, Chowdhurry HH, Zorec R, Kreft M (2015). Insulin and insulin-like growth factor 1 (IGF-1) modulate cytoplasmic glucose and glycogen levels but not glucose transport across the membrane in astrocytes. J. Biol. Chem..

[17] Mauras N (2000). Insulin-like growth factor I and growth hormone (GH) treatment in GH-deficient humans: Differential effects on protein, glucose, lipid, and calcium metabolism. J. Clin. Endocrinol. Metab..

[18] Castilla-Cortazar I (1997). mpaired intestinal sugar transport in cirrhotic rats: Correction by low doses of insulin-like growth factor I. Gastroenterology.

[19] Pascual M (2000). Altered intestinal transport of amino acids in cirrhotic rats: The effect of insulin-like growth factor-I. Am. J. Physiol.-Gastrointest. Liver Physiol..

[20] Smith WJ, Underwood LE, Clemmons DR (1995). Effects of caloric or protein restriction on insulin-like growth factor-I (IGF-I) and IGF-binding proteins in children and adults. J. Clin. Endocrinol. Metab..

[21] DeBoer MD (2017). Systemic inflammation, growth factors, and linear growth in the setting of infection and malnutrition. Nutrition..

[22] Aguayo V (2019). Fasciola hepatica GST downregulates NF-κB pathway effectors and inflammatory cytokines while promoting survival in a mouse septic shock model. Sci. Rep..

[23] Duff EMW, Anderson NM, Cooper ES (1999). Plasma insulin-like growth factor-1, type 1 procollagen, and serum tumor necrosis factor alpha in children recovering from Trichuris dysentery syndrome. Pediatrics.

[24] Tahapary DL (2017). Effect of anthelmintic treatment on insulin resistance: A cluster-randomized, placebo-controlled trial in Indonesia. Clin. Infect. Dis..

[25] Hassan AHI (1991). Circulating growth hormone, insulin-like growth factor I, cortisol and free thyroxine in children with schistosomiasis with and without hepatic fibrosis. J. Trop. Pediatr..

[26] Lunn PG, Northrop-Clewes CA (1993). Symposium on parasitism and protein and energy metabolism in man and animals. The impact of gastrointestinal parasites on protein-energy malnutrition in man. Proc. Nutr. Soc..

[27] Ling PR, Gollaher C, Colon E, Istfan N, Bistrian BR (1995). IGF-I alters energy expenditure and protein metabolism during parenteral feeding in rats. Am. J. Clin. Nutr..

[28] Soliman AT, Hassan AEH, Aref MK, Hintz RL, Rosenfeld RG, Rogol AD (1986). Serum insulin-Iike growth factors I and II concentrations and growth hormone and insulin responses to arginine infusion in children with protein-energy malnutrition before and after nutritional rehabilitation. Pediatr. Res..

[29] Shuto Y, Nakano T, Sanno N, Domoto H, Sugihara H, Wakabayashi I (1999). Reduced growth hormone receptor messenger ribonucleic acid in an aged man with chronic malnutrition and growth hormone resistance. J. Clin. Endocrinol. Metab..

[30] Wabitsch M, Heinze E, Debatin K-M, Blum WF (2000). IGF-l and IGFBP-3 expression in cultured human preadipocytes and adipocytes. Horm. Metab. Res..

[31] Gude MF, Frystyk J, Flyvbjerg A, Bruun JM, Richelsen B, Pederson SB (2012). The production and regulation of IGF and IGFBPs in human adipose tissue cultures. Growth Horm. IGF Res..

[32] Brismar K, Fernqvist-Forbes E, Wahren J, Hall K (1994). Effect of insulin on the hepatic production of insulin-like growth factor-binding protein-1 (IGFBP-1), IGFBP-3, and IGF-I in insulin- dependent diabetes. J. Clin. Endocrinol. Metab..

[33] Maizels R, Yazdanbakhsh M (2008). T-cell regulation in helminth parasite infections: Implications for inflammatory diseases. Chem. Immunol. Allergy..

[34] Mcsorley HJ, Maizels RM (2012). Helminth infections and host immune regulation. Clin. Microbiol. Rev..

[35] Warzecha Z (2003). IGF-1 stimulates production of interleukin-10 and inhibits development of caerulein-induced pancreatitis. J. Physiol. Pharmacol..

[36] Kooijman R, Coppens A (2004). nsulin-like growth factor-I stimulates IL-10 production in human T cells. J. Leukoc. Biol..

[37] Rosa BA (2018). Differential human gut microbiome assemblages during soil-transmitted helminth infections in Indonesia and Liberia. Microbiome..

[38] Yan J (2016). Gut microbiota induce IGF-1 and promote bone formation and growth. Proc. Natl. Acad. Sci..

[39] Easton AV (2019). The impact of anthelmintic treatment on human gut microbiota based on cross-sectional and pre- and post-deworming comparisons in Western Kenya. MBio..

[40] Audebert C (2016). Colonization with the enteric protozoa Blastocystis is associated with increased diversity of human gut bacterial microbiota. Sci. Rep..

[41] Solaymani-Mohammadi S, Genkinger JM, Loffredo CA, Singer SM (2010). A meta-analysis of the effectiveness of albendazole compared with metronidazole as treatments for infections with *Giardia duodenalis*. PLoS Negl. Trop. Dis..

[42] Bowers LW, Rossi EL, O’Flanagan CH, De Graffenried LA, Hursting SD (2015). The role of the insulin/IGF system in cancer: Lessons learned from clinical trials and the energy balance-cancer link. Front. Endocrinol..

[43] Tahapary DL (2015). Helminth infections and type 2 diabetes: A cluster-randomized placebo controlled SUGARSPIN trial in Nangapanda, Flores, Indonesia. BMC Infect. Dis..

[44] Kaisar MMM (2017). Improved diagnosis of *Trichuris trichiura* by using a bead-beating procedure on ethanol preserved stool samples prior to DNA isolation and the performance of multiplex real-time PCR for intestinal parasites. Parasitology.

