# Supplementary material for: Effect of anthelmintic treatment on serum free IGF-1 and IGFBP-3: a cluster-randomized-controlled trial in Indonesia
Source: Sci Rep. 2020 Nov 4;10:19023. doi: 10.1038/s41598-020-75781-4 (PMC7643058; doi:10.1038/s41598-020-75781-4)
Supplement: Supplementary file 1 — Supplementary Figures. [file 41598_2020_75781_MOESM1_ESM.pdf]

Supplementary Figures

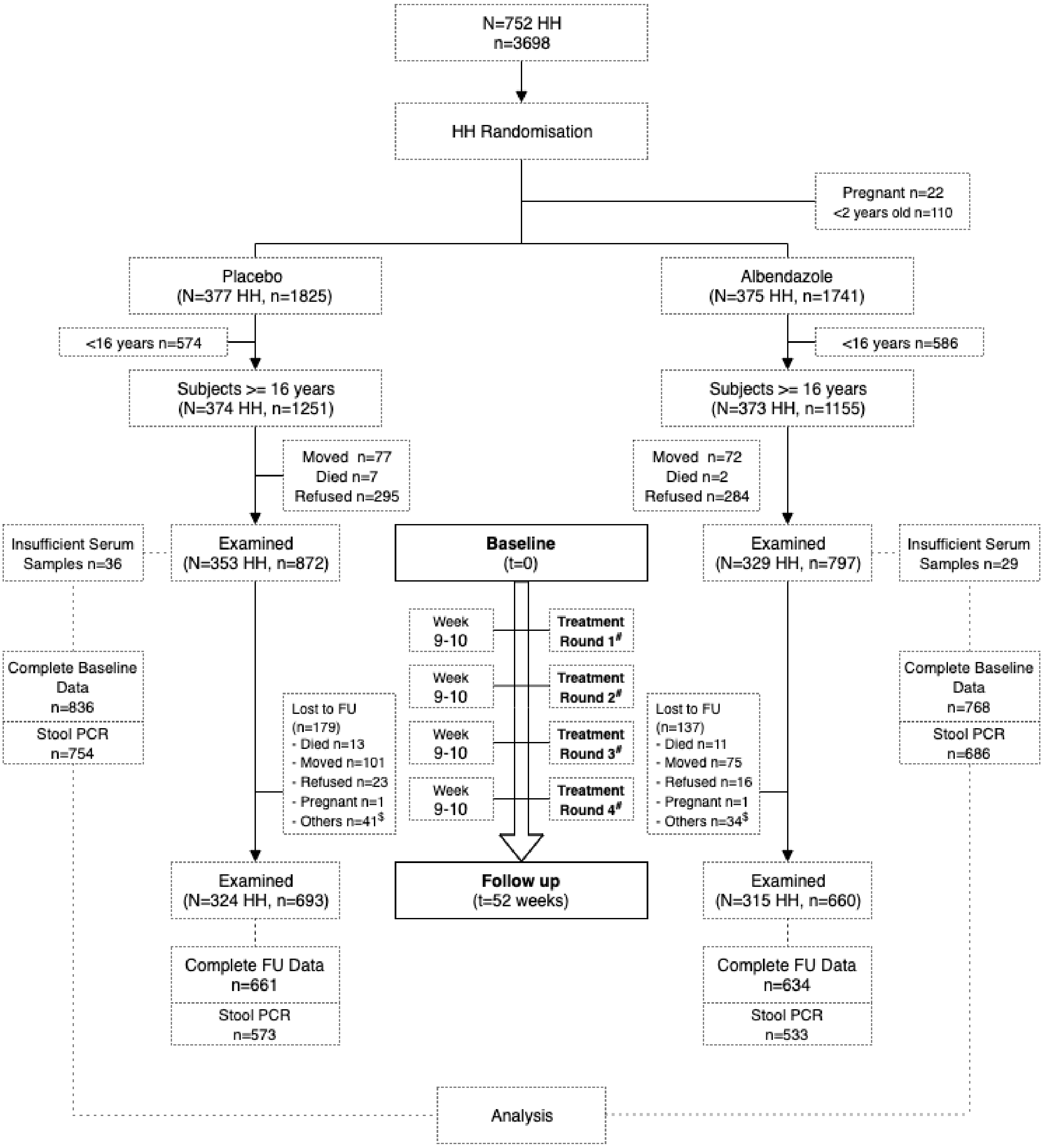

Supplementary Fig. 1 Consort Diagram

Baseline data (t=0) were collected during the first 8 weeks before the start of the drug administration. #Single dose of albendazole or matching placebo was given for three consecutive days to all household members, except children below 2 years of age and pregnant women. $Other reasons of loss to follow up were harvesting cops, working on funeral ceremonies, severely ill, hospitalized, nursing mother. HH: Household; FU: Follow Up.

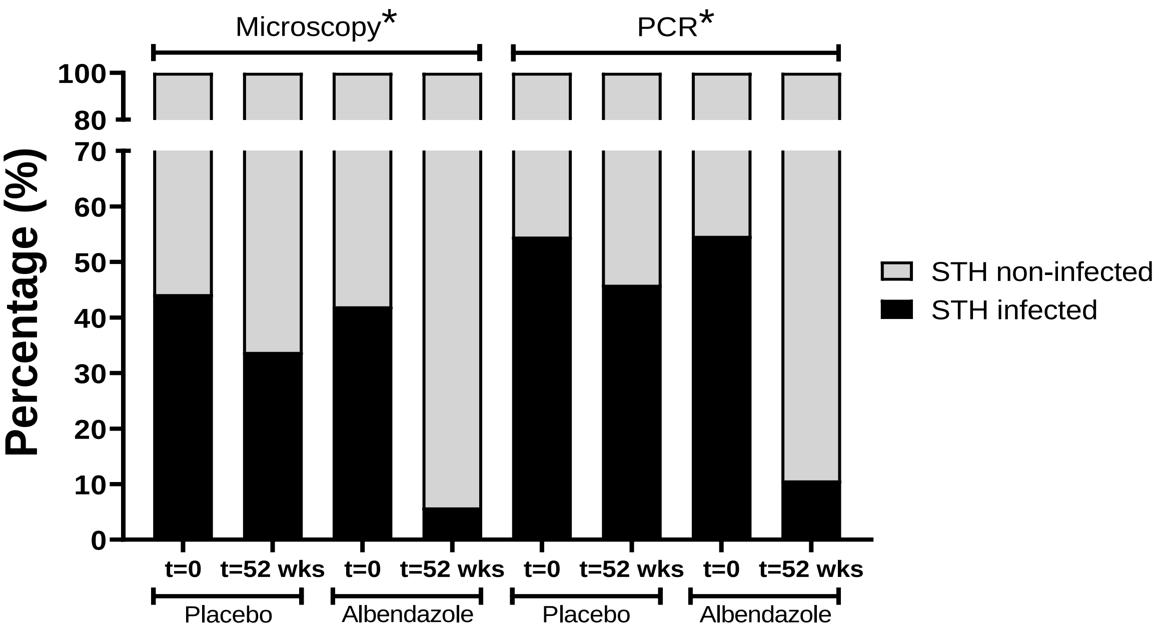

Supplementary Fig. 2 The effect of albendazole treatment on the prevalence of helminth infection.

Percentage of helminth infected subjects in the placebo and albendazole arms, as assessed by microscopy and polymerase chain reaction (PCR). p-values were calculated using a logistic model with random household effects and random subject effects.

*corresponds to P-value < 0.0001
